# Input device matters for measures of behaviour in online experiments

**DOI:** 10.1007/s00426-024-02065-1

**Published:** 2024-11-28

**Authors:** Matthew Warburton, Carlo Campagnoli, Mark Mon-Williams, Faisal Mushtaq, J. Ryan Morehead

**Affiliations:** 1 School of Psychology, University of Leeds, Leeds, UK; 2 Bradford Institute for Health Research, Bradford Hospitals National Health Service Trust, Bradford, UK; 3 National Centre for Optics, Vision and Eye Care, University of South-Eastern Norway, Kongsberg, Norway; 4 NIHR Leeds Biomedical Research Centre, Leeds, UK; 5 Centre for Immersive Technologies, University of Leeds, Leeds, UK

## Abstract

Studies of perception, cognition, and action increasingly rely on measures derived from the movements of a cursor to investigate how psychological processes unfold over time. This method is one of the most sensitive measures available for remote experiments conducted online, but experimenters have little control over the input device used by participants, typically a mouse or trackpad. These two devices require biomechanically distinct movements to operate, so measures extracted from cursor tracking data may differ between input devices. We investigated this in two online experiments requiring participants to execute goal-directed movements. We identify several measures that are critically influenced by the choice of input device using a kinematic decomposition of the recorded cursor trajectories. Those using a trackpad were slower to acquire targets, mainly attributable to greater times required to initiate movements and click on targets, despite showing greater peak speeds and lower variability in their movements. We believe there is a substantial risk that behavioural disparities caused by the input device used could be misidentified as differences in psychological processes. We urge researchers to collect data on input devices in online experiments and carefully consider and account for the effect they may have on their experimental data.

**Supplementary Information:**

The online version contains supplementary material available at 10.1007/s00426-024-02065-1.

Psychology studies conducted online have become a common part of an experimenter’s toolbox. While online experiments have been conducted for some time (Reips, 2001), the development of tools (Anwyl-Irvine et al., 2020; De Leeuw, 2015) and recruitment platforms (Crump et al., 2013; Palan & Schitter, 2018) have allowed more widespread adoption over the last decade, particularly during the COVID-19 pandemic where traditional laboratory-based testing paused for many. Online experiments allow large-scale, parallel recruitment of participants (Hartshorne et al., 2019) and the potential to reach a wide demographic (Gosling et al., 2010; Reinecke & Gajos, 2015). The compromise is that experimenters lose full control over testing, but many of the specific concerns around this seem acceptable, including data quality (Crump et al., 2013; Germine et al., 2012; Sauter et al., 2022; Tsay et al., 2021; Uittenhove et al., 2023) and temporal precision (Bridges et al., 2020; Chetverikov & Upravitelev, 2016; Reimers & Stewart, 2015). Online experiments appear to be a method that is here to stay, given this generally favourable trade-off.

Motor control and learning is an ideal testbed for online research methods. This area of inquiry has seen widespread adoption of online experiments to supplement or replace small-scale laboratory-based research (Avraham et al., 2022; Barradas et al., 2024; Cesanek et al., 2021; Coltman et al., 2021; Kim et al., 2022; Shyr & Joshi, 2024; Tsay et al., 2023; Wang et al., 2024a; Warburton et al., 2023a; Watral et al., 2023; Weightman et al., 2022). Some have also harnessed the promise of large-scale data collection, identifying predictors of visuomotor adaptation (Tsay et al., 2024) and investigating the long-term trajectory of learning in a first-person shooter game (Listman et al., 2021). This ready adoption is somewhat surprising because the movements executed by participants tend to be different to those measured in the laboratory. Most participants online will make movements with a computer mouse or trackpad, requiring relatively small movements of the wrist and fingers, whereas common laboratory-based experiments typically require larger arm movements through digitising tablets (Prablanc et al., 1975) or robotic arms (Morasso, 1981). Nevertheless, learning appears remarkably consistent in the laboratory and online in cases such as visuomotor adaptation (Tsay et al., 2021).

This reliance on standard computer peripheral devices aligns well with recent work utilising movements to infer details of processes beyond the movements themselves. While choices and reaction times (Donders, 1868/1969; Ratcliff, 1978; Thurstone, 1927) have been mainstay techniques to investigate perceptual and cognitive processing, they tend to only provide information about their outcome. The movements made towards different options can reveal discrete events, like changing one’s choice (Resulaj et al., 2009), but have seen great use in monitoring ongoing perceptual and cognitive processes (Dotan et al., 2019; Freeman et al., 2011; Koenig-Robert et al., 2023; Song & Nakayama, 2009; Spivey & Dale, 2006). This “mouse-tracking” technique (though it can be applied using any response modality that provides continuous movement input) has been applied to investigate a number of areas, such as lexical (Spivey et al., 2005) and numeric processing (Song & Nakayama, 2008), psychophysics (Bonnen et al., 2015), perceptual decision-making confidence (van den Berg et al., 2016), high-level cognitive decision-making (McKinstry et al., 2008), and working memory (Park & Zhang, 2024). This technique is also seeing use online (Ericson et al., 2021; Koenig-Robert et al., 2023; Kukona & Jordan, 2023; Li et al., 2023; Meidenbauer et al., 2023).

To make these inferences, spatiotemporal measures are extracted from the movements. For example, the curvature of a movement towards different competing options is taken to reflect conflict in the decision process (Song & Nakayama, 2009; Spivey et al., 2005). Properties like these have previously been shown to depend on the movement modality (Moher & Song, 2019), which poses a specific concern for online experiments as the input device used by participants cannot typically be controlled or restricted by the experimenter. This means that a typical online experiment will see a mixture of trackpad and mouse users who may show distinct movement properties, which can act as a potential confounder when making inferences about behaviour (Pronk et al., 2020; Reimers & Stewart, 2015). Where assessed, trackpads appear to be less efficient devices than computer mice in the context of regular computer use (Kar et al., 2015; Shanis & Hedge, 2003), requiring more time to execute goal-directed movements. Further, Fitts tasks have shown more specific differences between the devices, for example elevated reaction and movement times for trackpads (Hertzum & Hornbæk, 2010; MacKenzie et al., 2001). However, only a single study appears to have assessed input device differences in movement properties in the context of online behavioural research, finding elevated reaction times when using a trackpad (Watral et al., 2023).

In this study, we aimed to provide a rigorous assessment of how movements made with a trackpad and mouse differ online. We analysed movements made in two contexts: one where participants had to slice through targets, popular in studies of visuomotor adaptation (Avraham et al., 2022); and another where participants had to move to and click on a target, common in mouse-tracking studies (Dotan et al., 2019). In both contexts, we use participant movements to extract a number of spatiotemporal measures typically used to make inferences about behaviour, including reaction times, movement speeds, and the curvature of movement trajectories. Based on the available studies we expected that trackpads would show elevated temporal measures, like reaction times, but had no expectations related to spatial measures.

## Results

### Shooting movements

**Fig. 1:**
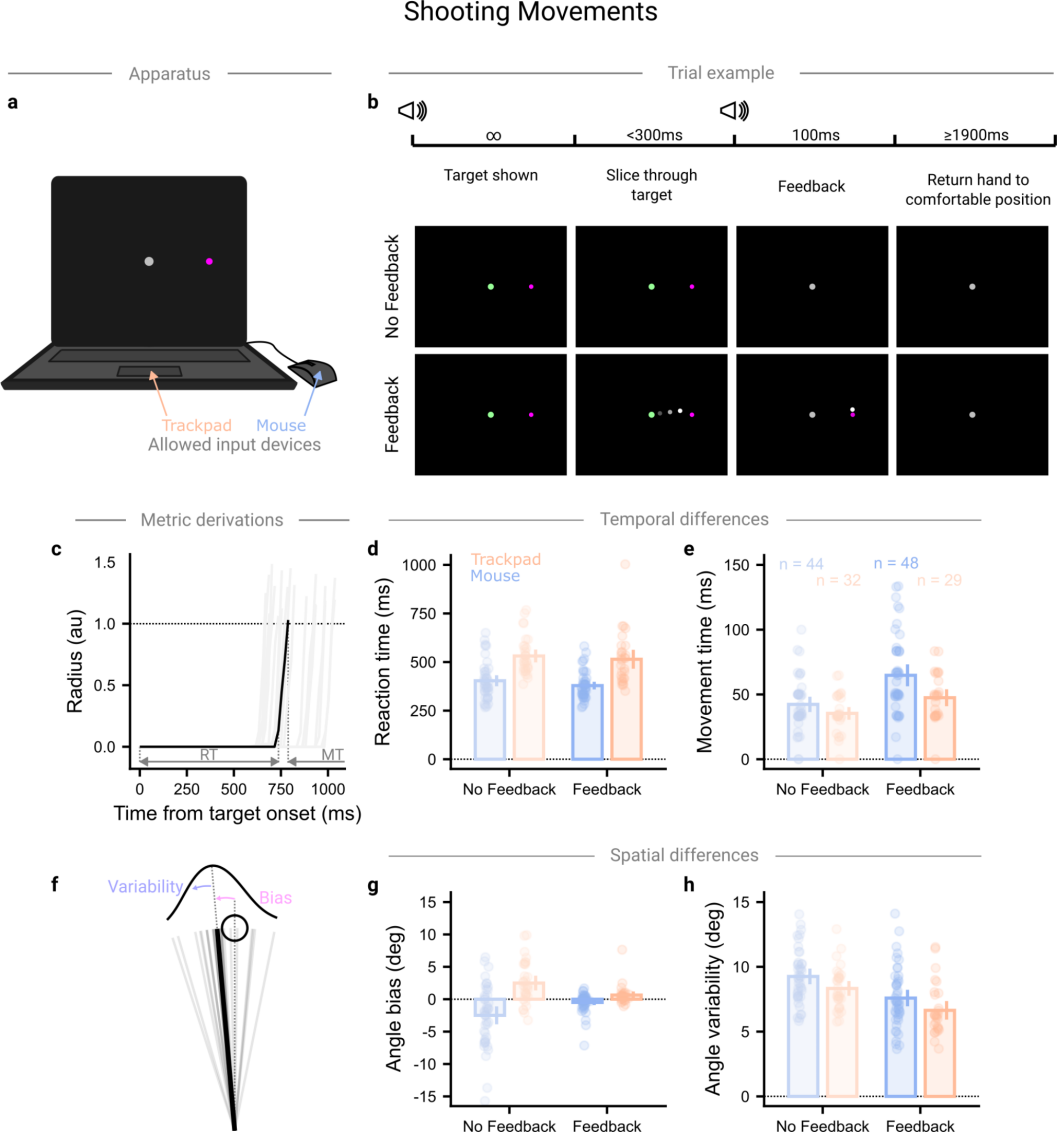
Analysis of shooting style movements. *Note*. (**a**) Participants completed the experiments on their own personal computer, and either used a trackpad or optical mouse to complete the experiment. (**b**) Participants were split into a group who did or did not have visual feedback of their reaches. Participants were required to execute single quick movements to slice through a presented target as accurately as they could. (**c**) Two temporal measures, reaction and movement times, were extracted from the hand movement data. (**d**) Reaction times were consistently higher for trackpad users, but (**e**) movement times were consistently lower. (**f**) Two spatial measures were also extracted from the movement data. The bias (**g**) gave the average end-point error, which was oppositely signed between devices and attenuated when feedback was available; and the end-point variability (**h**) was greater for mouse users in both feedback conditions

We first assessed differences between input devices in an experiment where participants had to make a single quick movement to slice through the target (often referred to as “shooting” or “slicing” movements). Participants completed the experiment online and used their own computer setup, meaning a mixture of mouse and trackpad users completed the experiment (Fig. 1a). Participants were split into two groups who either did or did not receive visual feedback of their reaches (Fig. 1b). We included this manipulation because studies utilising shooting movements often preclude feedback during certain parts of the experiment. For those in the Feedback group, online feedback of the cursor was shown during the reach, as well as end-point feedback at the target radius, whereas participants in the No Feedback group received neither. After a reach, participants moved their hand back to a comfortable position in the workspace, the cursor was reset to screen centre, and a new target was shown. Participants completed 480 trials, arranged into cycles of 24 targets where each equally-spaced target direction between 0° and 345° was tested once in a pseudo-randomised order.

We first investigated two temporal measures - the reaction time, between a target being shown and a reach being initiated; and the movement time, between motion onset and the target radius being passed (Fig. 1c). This was done through mixed ANOVAs with a between-subject effect (input device; Mouse or Trackpad) and a within-subject effect (feedback condition; Feedback or No Feedback). There was a clear effect of input device on reaction times (Fig. 1d), with mouse users showing significantly shorter reaction times than trackpad users (Mouse: 391 ms [368 ms, 414 ms], Trackpad: 523 ms [495ms, 551 ms]; $\:{F}_{1,149}=67.43,{\eta\:}_{g}^{2}=0.31,p<.001$). There was no significant main effect of feedback condition ($\:{F}_{1,149}=1.70,{\eta\:}_{g}^{2}=0.01,p=.194$) or interaction ($\:{F}_{1,149}=0.06,{\eta\:}_{g}^{2}<0.01,p=.800$). The opposite effect was true for movement times (Fig. 1e), with mouse users showing significantly longer movement times than trackpad users (Mouse: 54 ms [48 ms, 59 ms], Trackpad: 41 ms [35 ms, 48 ms]; $\:{F}_{1,149}=10.79,{\eta\:}_{g}^{2}=0.07,p=.001$). Further, those in the Feedback group showed longer movement times than those without feedback (Feedback: 56 ms [50 ms, 62 ms], No Feedback: 39 ms [33 ms, 45 ms]; $\:{F}_{1,149}=21.92,{\eta\:}_{g}^{2}=0.13,p<.001$), with no significant interaction ($\:{F}_{1,149}=1.98,{\eta\:}_{g}^{2}=0.01,p=.161$).

We next investigated spatial aspects of the movement and focus on the bias and variability (Fig. 1f) in the hand angle at the target radius. Mouse and trackpad users showed oppositely signed biases that were attenuated when feedback was available (Fig. 1g). A main effect of input device ($\:{F}_{1,149}=35.60,{\eta\:}_{g}^{2}=0.19,p<.001$) confirmed an overall difference in bias, while there was no main effect of feedback condition ($\:{F}_{1,149}=0.02,{\eta\:}_{g}^{2}<0.01,p=.896$). There was a significant interaction between the two ($\:{F}_{1,149}=14.04,{\eta\:}_{g}^{2}=0.09,p<.001$), with follow-up comparisons showing there was a significant difference in bias between input devices when feedback was not available (Mouse: -2.5° [-3.4°, -1.6°], Trackpad: 2.5° [1.4°, 3.6°]; $\:{t}_{149}=6.91,p<.001$) but no significant difference when feedback was available (Mouse: -0.5° [-1.4°, 0.4°], Trackpad: 0.6° [-0.5°, 1.8°]; $\:{t}_{149}=1.56,p=.121$). The variability of movements was influenced by input device (Fig. 1h), with mouse users showing higher variability than trackpad users (Mouse: 8.4° [8.0°, 8,9°], Trackpad: 7.5° [6.9°, 8.1°]; $\:{F}_{1,149}=7.87,{\eta\:}_{g}^{2}=0.05,p=.006$). The availability of feedback also influenced variability, with greater variability when no visual feedback was available (Feedback: 7.1° [6.6°, 7.7°], No Feedback: 8.8° [8.3°, 9.3°]; $\:{F}_{1,149}=25.31,{\eta\:}_{g}^{2}=0.15,p<.001$). There was no significant interaction ($\:{F}_{1,149}=0.00,{\eta\:}_{g}^{2}<0.01,p<.973$). For both variables, we also found input device-related differences across movement directions (see Supplementary Fig. 1).

**Fig. 2:**
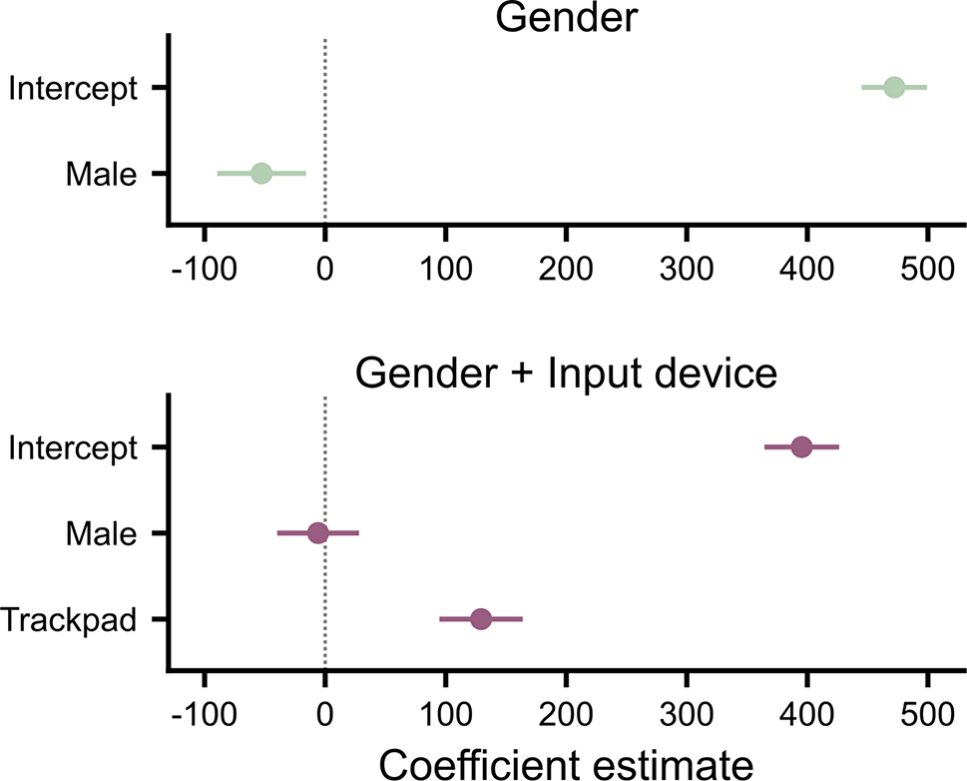
Artefactual gender differences in reaction times in the shooting movement experiment. *Note*. When only gender was entered as an independent variable in the linear regression, males showed significantly faster reaction times than females (top panel). When gender and input device were entered as independent variables, there is no significant (or meaningful) difference between males and females, with a large difference between mouse and trackpads (bottom panel). Points show the coefficient estimate associated with each variable, and error bars show 95% confidence intervals

The reaction time analysis provides a demonstration of the risk that arises when input device is not accounted for (Fig. 2). In an exploratory analysis, a linear regression showed that males had significantly faster reaction times than females ($\:{\beta\:}_{Male}=-53\hspace{0.25em}\text{ms}\hspace{0.25em}\left[-90\hspace{0.25em}\text{ms},-16\hspace{0.25em}\text{ms}\right],{t}_{149}=-2.83,p=.005$), but this relationship disappeared when input device was included in the regression ($\:{\beta\:}_{Male}=-6\hspace{0.25em}\text{ms}\hspace{0.25em}\left[-40\hspace{0.25em}\text{ms},28\hspace{0.25em}\text{ms}\right],{t}_{148}=-0.34,p=.733$; $\:{\beta\:}_{Trackpad}=129\hspace{0.25em}\text{ms}\hspace{0.25em}\left[95\hspace{0.25em}\text{ms},164\hspace{0.25em}\text{ms}\right],{t}_{148}=7.40,p<.001$). This confounding effect arose because of a significant difference in the user’s gender per input device, where more males used a mouse and more females used a trackpad (see Methods). This demonstrates the risk of not accounting for the input device used. The same analysis was checked for the other spatiotemporal variables but no others showed the same counterfactual gender effect.

### Point-to-point movements

**Fig. 3:**
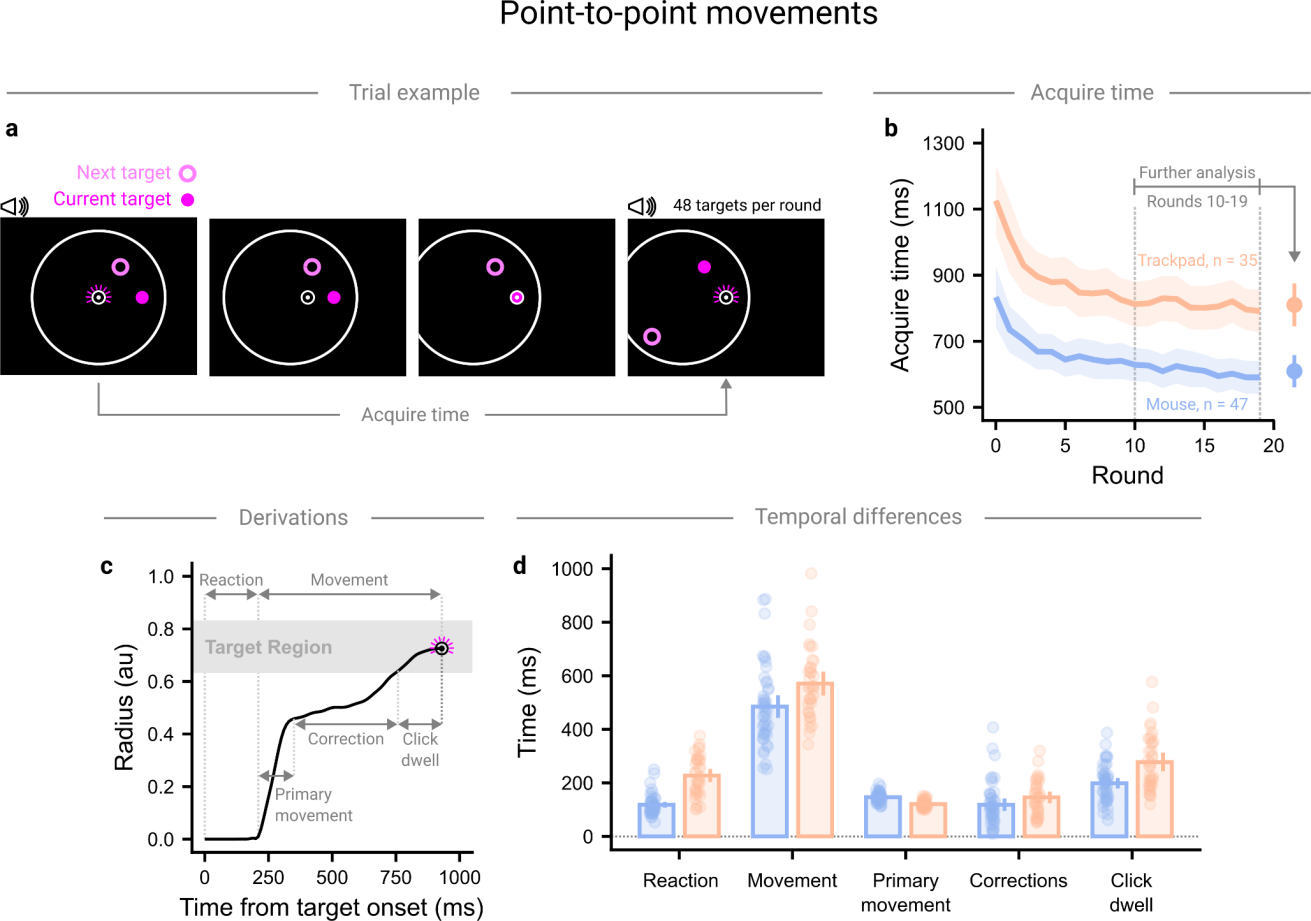
Temporal analysis of point-to-point style movements. *Note*. (**a**) Participants completed 20 rounds of movements to 48 targets. During a trial, participants could see the current and next target. Participants were required to move to and click upon the current target, upon which the next target changed to the current target and a new next target was revealed. (**b**) The acquire time, the total time between successive successful shots, improved rapidly over the first 5 rounds before stabilising. Trackpad users showed greater acquire time across the experiment. (**c**) Acquire time was broken down into further temporal variables. (**d**) Trackpad users showed greater reaction and movement times. Movement time was further decomposed, with trackpad users showing greater click dwell times but lower primary movement times. Points show participant averages, solid lines and bars show group averages and shaded regions and vertical lines show 95% confidence intervals

We next turned to point-to-point movements, which more closely resemble the types of movements typically studied in mouse-tracking paradigms, to further assess differences between input devices. To do this, we re-analysed a previously published dataset (Warburton et al., 2023a) where participants used their own computer setup to complete an online experiment inspired by FPS aim-training games. Participants completed 20 rounds of the task. Each round consisted of participants moving to and clicking on 48 targets, arranged in seemingly random patterns, as quickly as they could (Fig. 3a). Within each round, each combination of 8 target angles (0° to 315° in 45° increments) and 3 movement distances (0.4 au, 0.6 au, 0.8 au) was tested twice, though the presented analyses averaged over these variables.

We assessed the acquire time - the total time between two consecutive successful shots (Fig. 3b) - using a mixed ANOVA, with a within-subject effect (round) and a between-subject effect (input device; Mouse or Trackpad). Mouse users were faster than trackpad users throughout ($\:{F}_{1,79}=24.58,{\eta\:}_{g}^{2}=0.20,p<.001$). Participants using both input devices improved their performance over the 20 rounds ($\:{F}_{2.56,202.60}=45.07,{\eta\:}_{g}^{2}=0.10,p<.001$), with no significant evidence that either group improved more than the other ($\:{F}_{2.56,202.60}=2.18,{\eta\:}_{g}^{2}=0.01,p=.102$). We decided to restrict all further analyses to only movements during the last 10 rounds, where performance had become more stable. During these last 10 rounds, mouse users were significantly faster than trackpad users (Mouse: 609 ms [560 ms, 658 ms], Trackpad: 810 ms [745 ms, 875 ms]; $\:{t}_{80}=4.94,p<.001$).

We used features of the movement to break the acquire time down into meaningful periods of time (Fig. 3c). In particular, for each movement we calculated the reaction time (the time between a new target becoming active and a movement initiated towards it) and the movement time (the time between movement initiation and the target being clicked). Mouse users were significantly quicker than trackpad users during both the reaction (Mouse: 118 ms [107 ms, 129 ms], Trackpad: 228 ms [202 ms, 253 ms]; $\:{t}_{80}=8.53,p<.001$) and movement phases (Mouse: 485 ms [443 ms, 527 ms], Trackpad: 571 ms [526 ms, 616 ms]; $\:{t}_{80}=2.71,p=.008$) of the trial (Fig. 3d).

The movement time could be further decomposed into the primary movement time (the time between a movement being initiated and the end of the ‘ballistic’ phase of a movement), the correction time (the time required to execute feedback-driven corrections to bring the cursor into the target), and the click dwell time (the time between the target being entered and a click being registered). Mouse users showed significantly quicker click dwell times (Mouse: 199 ms [179 ms, 219 ms], Trackpad: 278 ms [243 ms, 313 ms]; $\:{t}_{80}=4.11,p<.001$). Trackpad users reached the end of the primary movement sooner than mouse users (Mouse: 147 ms [141 ms, 152 ms], Trackpad: 121 ms [116 ms, 126 ms]; $\:{t}_{80}=6.87,p<.001$), with no significant difference during the correction phase of the movement (Mouse: 118 ms [95 ms, 141 ms], Trackpad: 146 ms [124 ms, 168 ms]; $\:{t}_{80}=1.65,p=.103$).

**Fig. 4:**
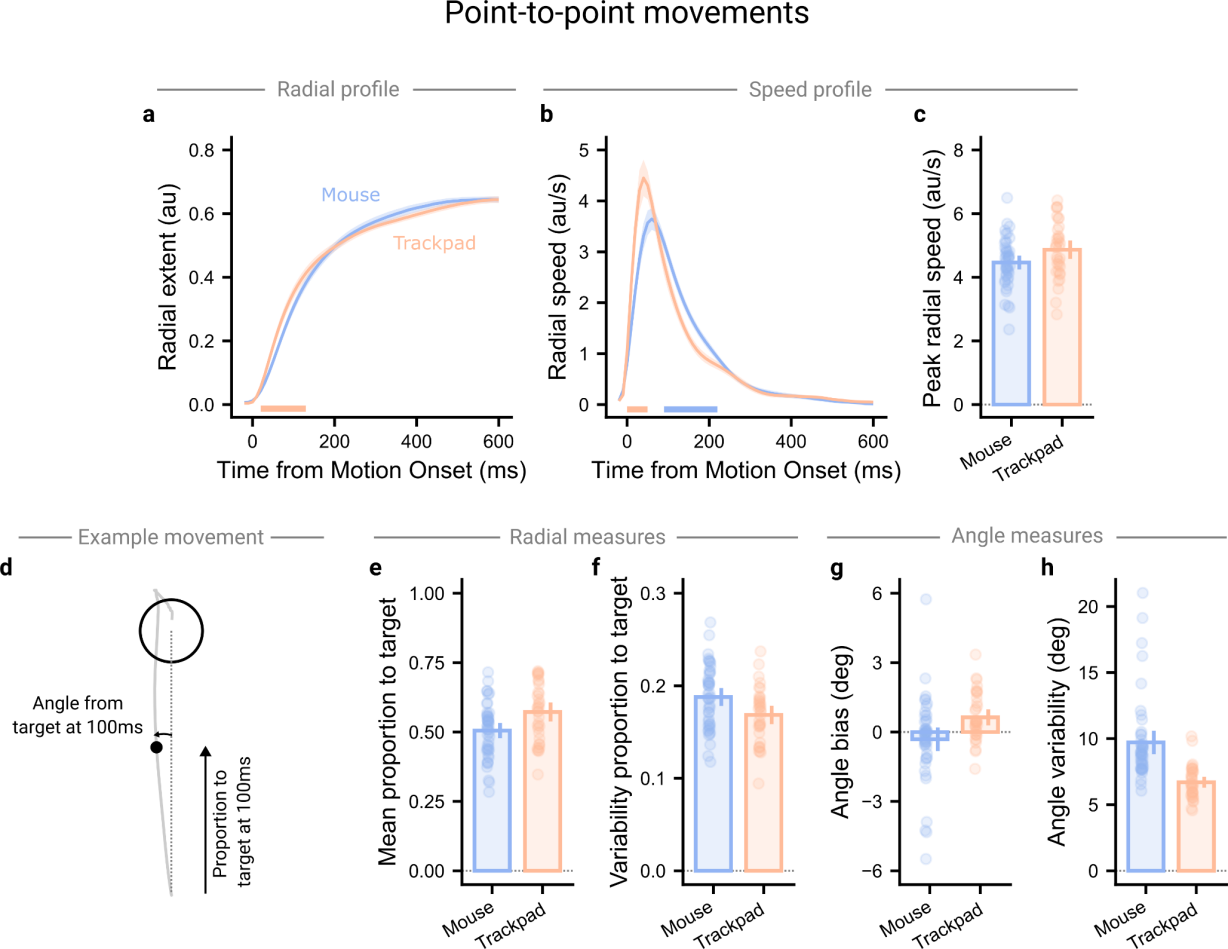
Spatial analysis of point-to-point style movements. *Note*. (**a**) Trackpad users reached a greater radial extent earlier into their movements but were not significantly different later in the movement. (**b**) This was reflected in radial speed, where trackpad users moved more quickly the start of the movement but slower later in the movement. (**c**) The peak radial speed reached during each movement was extracted, showing trackpad users had greater peak radial speeds. Note the peak speeds here are greater than the peak speed reflected in panel (**c**) because peak speed could be reached at different times across trials, resulting in a smoothed but lower peak speed for the continuous function. (**d**) Two spatial measures were extracted from cursor data at 100ms into the movement, where the difference in radial extent appeared to be roughly maximised - the proportion of the target distance covered and the angular offset from the target. (**e**) Trackpad users covered a greater proportion of the distance to the target at 100ms after motion onset, and (**f**) showed less variability in the proportion of the distance covered. (**g**) Trackpad and mouse users showed small, oppositely signed biases, but (**h**) mouse users showed substantially greater variability in angle from the target at 100ms into the movement. Points show participant averages, solid lines and bars show group averages, shaded regions and vertical lines show 95% confidence intervals, and horizontal lines show clusters of significant differences

We next focussed on spatial aspects of the movements. Trackpad users reached a greater radial extent early in the movement (between 20 and 130 ms after motion onset, $\:p=.011$), but both input devices converged thereafter (Fig. 4a). Trackpad users showed greater radial speed immediately after motion onset (between 0 and 50 ms, $\:p=.008$, but were then slower than mouse users after peak speed had been reached (between 90 and 220 ms, $\:p<.001$), converging after this (Fig. 4b). The speed profile for trackpad users appeared qualitatively less smooth than for mouse users, with an additional bump in the profile around 200 ms. This was reflected in the log dimensionless jerk metric, where values closer to zero reflect a smoother movement (Mouse: -9.2 [-9.5, -9.0], Trackpad: -10.4 [-10.7, -10.1]; $\:{t}_{80}=5.56,p<.001$). The peak speed reached during the movements was greater for trackpad users (Mouse: 4.46 au/s [4.25 au/s, 4.68 au/s], Trackpad: 4.87 au/s [4.58 au/s, 5.16 au/s]; $\:{t}_{80}=2.23,p=.029$; Fig. 4c). We note that these continuous functions are, in some ways, misleading. While the early ‘ballistic’ phase of the movement is fairly well stereotyped, and as such the functions should be representative of the early portion of an average movement, because different participants take different amounts of time to complete their movements, the number of participants captured by the continuous function will decrease with time from motion onset. The functions, therefore, do not necessarily reflect any particular set of participants, or even trials, well.

We therefore extracted further measures at a specific timepoint where all trials should be captured, 100 ms after motion onset. This timepoint is also early enough that the extracted measures should represent differences during the “ballistic” phase of the movement. We focussed on average and variability in hand angle, as well as the proportion of the distance covered in the direction of the target (Fig. 4d). In alignment with the radial extent profile, trackpad users had made more progress towards the target by 100ms after motion onset (Mouse: 0.51 [0.48, 0.53], Trackpad: 0.57 [0.54, 0.61]; $\:{t}_{80}=3.03,p=.003$; Fig. 4e), and did so more consistently than mouse users (Mouse: 0.19 [0.18, 0.20], Trackpad: 0.17 [0.16, 0.18]; $\:{t}_{80}=2.69,p=.009$; Fig. 4f). Consistent with the observations from the shooting analysis, both input devices showed small but oppositely signed angular biases (Mouse: -0.31° [-0.82°, 0.20°], Trackpad: 0.64° [0.29°, 0.98°]; $\:{t}_{80}=2.81,p=.006$; Fig. 4g), and mouse users showed greater angular variability (Mouse: 9.7° [8.8°, 10.6°], Trackpad: 6.7° [6.3°, 7.1°]; $\:{t}_{80}=5.43,p<.001$; Fig. 4h). This elevated angular variability is corroborated by the linearity index, where mouse users showed movements that deviated further from the straight line between the movement start point and the target centre (Mouse: 0.079 [0.073, 0.085]; Trackpad: 0.053 [0.048, 0.058], $\:{t}_{80}=6.17,p<.001$).

## Discussion

Despite an increasing number of studies investigating movements made by individuals online, how these movements are influenced by the input device used has received little attention. We conducted kinematic analyses of mouse and trackpad movements made towards targets by participants in two online experiments. Targets could be acquired more rapidly by participants using a mouse, primarily driven by shorter times required to initiate movements and click on a target once in a position to do so, despite trackpad users moving faster initially and executing movements with lower spatial variability. This highlights input device driven differences in movement metrics that are used to infer details of a range of perceptual, cognitive, and motoric processes. There is a risk that, if unaccounted for, these input device differences could be mistaken for behavioural differences. For example, reaction times in the shooting movement experiment show a significant relationship with gender that disappeared when controlling for input device.

### Differences in movement properties

We focussed on a number of temporal and spatial metrics that are commonly used to understand the processes underlying behaviour. Here we find that reaction times were consistently higher when using a trackpad, both in shooting and point-to-point movements. This agrees with previous studies that found a similar difference in reaction time between devices (Hertzum & Hornbæk, 2010; Watral et al., 2023). While we cannot rule out that differences in cognitive processing demands contribute to this (Watral et al., 2023), we believe a combination of device latency (e.g. trackpad movements may be handled by the computer with a greater delay) and physiology (e.g. users may choose to hover their finger above the trackpad until a movement is required) is most likely to explain this difference. Elevated click dwell times for trackpad users supports this conjecture, as we might not expect the button pressed to elicit substantially different processing demands, but could be subject to different input delays and physiological demands.

We also found that trackpad users showed lower movement times for shooting movements. During these movements, participants typically intercept the target close to peak speed (e.g. Wang et al., 2024a), so the lower movement times are consistent with the greater peak speeds found for trackpad users in point-to-point movements (Fig. 4b). The difference in peak speeds reached between the devices could be related to the respective inertial properties of the movements. While using a computer mouse relies on movements of the fingers, wrist, elbow, and shoulder (Kang et al., 2024), the shoulder and elbow are relatively more fixed in trackpad use (Conte, 2014). This may also explain the difference in primary movement time found in the point-to-point movements, as the lower inertia of movements of the lower arm during trackpad use may allow an initial correction to be initiated more readily. In contrast, movement times were greater for trackpad users in the point-to-point movements. While this diverges from Watral et al. (2023), who found no significant difference in movement time between the devices, their task did not require participants to click inside a target to terminate a trial, whereas the click dwell phase accounted for the bulk of the movement time difference observed here.

We also found differences in the spatial properties of the cursor movements between input devices. In both tasks we found oppositely signed angular biases (and differences in the pattern of bias across direction, Supplementary Fig. 1) that were amplified when feedback was withheld in the shooting movement experiment. While the reason that angular biases arise is still debated (Wang et al., 2024b), the difference between the devices might reflect postural differences. While trackpads are typically placed along the body mid-line, computer mice are typically held to the right of the mid-line (Conte, 2014; Lin et al., 2015). When forced to adopt similar postures in arm reaching (Ghilardi et al., 1995), the posture closest to mouse use had a more negative average bias, consistent with those observed here. Further, we also found that trackpad users showed lower angular variability and movement curvature than mouse users, consistent with previous work (Hertzum & Hornbæk, 2010). One possibility is that this is also related to the inertial properties of the physical movements, whereby the greater recruitment of the upper joints of the arm in mouse movements may result in greater signal-dependent noise (Harris & Wolpert, 1998). Further work that measures the physical movements executed during mouse and trackpad use could provide greater clarity on the relationship between physical and task-space movements.

From the current work, it is unclear how the user’s experience with their input device might mediate the differences observed between the two. Participants accessed the experiment in their own time, presumably using computer hardware that they were comfortable with, so we expect that participants had ample experience with the input device they used to complete the experiment. Further, while we observed rapid improvements in acquire time over the point-to-point movement experiment, this improvement was not significantly greater for either users. Nevertheless, the ubiquity of the computer mouse may mean the average user has more experience with it, which may partly account for the differences observed. Even with additional experience, if many of the differences observed are indeed due to fundamental technological and physiological differences between the devices, we would expect persistent differences to remain.

### Implications for online research

Generally one cannot control the input device used when recruiting from online participant pools or citizen science websites, with a fairly equal split between users (e.g. Tsay et al., 2024). Given the differences in movement properties observed, experimenters should collect information on the input device used by participants in online experiments and account for how it might influence the collected data. Measures similar to those studied here are used to infer properties of the processes underlying behaviour. The curvature of movements towards different options is used as a measure for choice conflict (Song & Nakayama, 2009), the onset of trajectory deviations indicating the timing of underlying processes (Sullivan et al., 2015), the speed of movement reflecting choice confidence (Dotan et al., 2018), and movement properties at peak speed often used to investigate motor learning (Morehead et al., 2015). There is, therefore, a risk that unaccounted-for device-dependent differences in movement properties could confound certain inferences. How these effects manifest could be counter-intuitive – a combination of greater input delay and movement speed on trackpads could give rise to greater curvature in a typical mouse-tracking choice task, despite observing lower curvature here in simple point-to-point movements – and will depend on the exact experiment conducted.

It is worth noting that the risk posed by the input device used is not restricted to the online platform. In the laboratory, we would generally expect a given study to utilise a consistent experimental setup. The risk of confounding in this scenario arises when cross-experimental comparisons are made where both a variable of interest and the input device utilised has changed, such that any behavioural change attributed to the former may actually be caused by the latter. A similar risk is present online, where two experiments may have different ratios of input devices used, but the reaction time analysis in the shooting movement experiment shows the risk of confounding is present even within a single experiment.

One of the main concerns with conducting experiments online is that the hardware and software used by participants cannot be controlled. A major point of contention has been the temporal precision available in online experiments (Anwyl-Irvine et al., 2021; Bridges et al., 2020; Chetverikov & Upravitelev, 2016; Reimers & Stewart, 2015). While temporal precision in stimuli onset time, stimuli display time, and reaction times are affected by factors like the type of computer device, the web browser, and the experimental framework used (Anwyl-Irvine et al., 2021; Bridges et al., 2020), in general these have only a modest effect on all but the most temporally-sensitive experiments (Pronk et al., 2020; Reimers & Stewart, 2015). The risks posed by temporal precision are greatest for studies of individual differences and can be mitigated to some extent by using within-subject designs (Pronk et al., 2020).

Similarly, the importance of device-dependent differences in movement properties will depend on details of the experiment. It is likely that using within-subject designs will, in part, mitigate these risks. For example, here we found that the *improvement* in acquire time with practice was similar between input devices despite differences in *absolute* acquire time. However, given the variety of ways that movements are used, there will be no magic bullet for eliminating the issue. In certain cases, these differences may not be important. For example, where assessed, the input device used has not been found to significantly influence visuomotor adaptation (Kim et al., 2022; Tsay et al., 2021, 2024; Warburton et al., 2023b; Watral et al., 2023), though more research would be required to assert this across the variety of manipulations used in adaptation research. In contrast, here we found that properties of the movements themselves differed greatly in ways that may be important for mouse-tracking studies. As such, the onus will be on experimenters to account for this potentially confounding variable within their specific experiments.

## Method

### Participants

Participants for the shooting movement experiment were recruited through the online recruitment platform Prolific, restricted to those living in the UK or USA, who had English as a first language, and had a Prolific approval rating of 95% or above, and were paid £3.75 upon completion. The experiment was approved by the School of Psychology Ethics Committee at the University of Leeds, and participants gave informed consent via a Qualtrics web form prior to starting the study. The data used in the point-to-point analyses have previous been published (Warburton et al., 2023a). No participants completed both experiments.

For the shooting movement experiment, we excluded 7 (out of 160) participants who we deemed did not complete the experiment as intended, which we operationalised as where more than 20% of their trials had any of the following issues: they failed the trial by moving too slowly; their reach error was more than 60° either side of the target; and their reach error was more than 40° away from a rolling median of recent reach errors. This left 153 participants remaining, who reported a mixture of mouse and trackpad use (Mouse: *n* = 92, mean ± SD age = 41 ± 13, male = 63, female = 28; Trackpad: *n* = 61, mean ± SD age = 38 ± 11, male = 19, female = 41). We note that the proportion of male and female users per device was significantly different for this experiment. We conducted additional analysis controlling for gender (see Data processing and statistical analyses) but found none of the input-device effects differed with its inclusion.

For the point-to-point movement experiment, we used the same exclusion criteria as detailed in the original paper (Warburton et al., 2023a), but additionally excluded 4 participants whose reach error 100ms after motion onset was more than 60° either side of the target on more than 20% of trials. This left 82 participants remaining, who reported a mixture of mouse and trackpad use (Mouse: *n* = 47, mean ± SD age = 44 ± 13, male = 25, female = 21; Trackpad: *n* = 35, mean ± SD age = 41 ± 13, male = 16, female = 19).

### Apparatus

Participants used their own personal computers to complete the experiments, restricted to desktops and laptops using Prolific’s screening tool. The experiments were built using Unity (version 2019.4.15f) and the Unity Experiment Framework (Brookes et al., 2019), delivered via a WebGL build on a website, and uploaded data to a remote database. Stimuli were not scaled to a constant size, so the physical size of the task differed depending on the screen size used by participants. However, the task always took up the full height of the screen and was designed to be visible on a 4:3 monitor, with wider monitors featuring more task-irrelevant background textures. Therefore, the size of the task relative to the screen height was consistent across hardware. Progress could only be made with the task in full-screen mode, and the desktop cursor was hidden and locked so it could not be used to interact with the web page. Instead, the raw mouse or trackpad input was used to perform in-game movements of the cursor (i.e. operating system mouse acceleration profiles were disabled, a common recommendation for mouse-tracking studies; Fischer & Hartmann, 2014). The sensitivity of in-game movements was calibrated to be similar to the participants’ desktop cursor.

### Experimental task and procedure

In both experiments, participants first completed a form to provide demographic information, including the input device they were using, before pressing buttons to check they could hear the game audio and to progress to the experiment. Participants used their usual desktop cursor during this stage to navigate the form while the cursor movements were monitored in pixel and Unity game space, which provided an initial calibration so that in-game movements had similar sensitivity to their usual computer use. Participants then progressed to a tutorial that gradually introduced elements of the relevant experiment so that they understood the task requirements. In the experiment assessing point-to-point movements, participants then had a chance to adjust the sensitivity of their in-game cursor to their preferred level before starting the experimental trials.

#### Shooting movement experiment

Participants then started the main experimental trials. Participants were split into two groups, who either did or did not receive visual feedback during their reaches. An experimental trial for those who did not receive visual feedback of their reach is explained first. During a trial, a start-point (diameter = 4% screen height) was always visible in the centre of the screen. On trial start, a low beep sounded, the start-point turned green, and a magenta target (diameter = 2.5% screen height) was revealed. Participants had to make a single quick “shooting” movement through the target. On a successful trial, upon reaching the target radius, participants were given neutral feedback (a higher beep sound, around 100 ms in duration) to indicate they had made a reach of a great enough distance within a 300 ms time limit. On trials where participants took longer than 300 ms to reach the target radius, an aversive buzzing sound played instead of the neutral beep, and text feedback was shown for 2 s relaying the time they took to move, to act as a deterrent. Following a reach, participants had to return their hand to a comfortable position within the workspace. A new trial would begin once it had been at least 1900 ms since feedback was given (1500 ms on error trials) and participants had been stationary for at least 300 ms, to provide enough time to do this procedure comfortably. When the next trial started, the in-game cursor’s position was reset to the centre of the screen. During no feedback trials, a graphical representation of the in-game cursor was never shown to participants.

When feedback was provided, the in-game cursor (white circle, diameter = 2.5% screen height) was shown upon a trial starting. Participants were given online cursor feedback until the target radius had been crossed. On the frame where the target radius was crossed, the cursor position on the current and previous frames were used to interpolate the cursor’s position at the target radius, and end-point feedback was provided for 100 ms, where the cursor position was frozen at this interpolated position while the target remained on the screen. After this end-point feedback, the cursor and target disappeared, and participants had to return their hand to a comfortable position without feedback, as in a no feedback trial.

Both groups completed 20 blocks of 24 trials (480 trials total). Within each block, the target was shown at each angle in the set {0°, 15°, 30°,…, 345°} once in a random order (with 0° being directly right of the start-point, and increasing angles going counter-clockwise). Targets were always located on an imaginary circle, with a diameter of 80% of the screen height. Movements, therefore, had an index of difficulty (Shannon formulation; Soukoreff and MacKenzie (2004) of 4.09 bits. Participants were given an untimed break every 4 blocks. After completing the experimental trials, participants were given information on their average error over the experiment and were then returned to Prolific.

#### Point-to-point movement experiment

Details of experimental trials and procedures for this experiment have been described previously (Warburton et al., 2023a), so we provide only a brief description here. Instead of mouse or trackpad movements translating a cursor across the screen, this experiment used “Mouselook” style visual feedback, where movements were used to pan the view of a camera in the scene. This is consistent with visual feedback shown in first-person shooter games, and we previously found movements differ little between “Mouselook” and more traditional “Pointing” style visual feedback (Warburton et al., 2023a). A start-point was visible in the middle of the screen. Upon moving to and clicking upon it, the start-point disappeared, the current target was shown (solid magenta circle) and the following target was also shown (hollow magenta circle). Participants were required to move to and click on the current target. Upon successfully clicking on the current target, the previous next target became the current target (the hollow area filled in) and a new following target was revealed. Participants continued this until 48 targets had been clicked. Movements had no time limit, but an on-screen timer visible during the trial encouraged participants to complete the rounds as quickly as possible.

Participants completed 20 rounds, with each 48-target round consisting of two uninterrupted cycles. Targets could be located at one of three distances {0.4 au, 0.6 au, 0.8 au} and one of eight target directions {0°, 45°,…, 315°} from the previous target or start-point, with each combination tested once per cycle. Movement index of difficulty in this experiment ranged between 1.58 and 2.32 bits. Each target sequence was generated by simulating new sequences until one was found where no target was more than 1 au from the workspace centre. This gave seemingly random target sequences while controlling the statistics of the sequences. Participants were given an untimed break between each round. Following the experiment, participants completed a questionnaire before being returned to Prolific.

### Data processing and statistical analyses

All data processing and statistical analyses were performed using custom R scripts (version 4.2.2). The statistical significance threshold was set at *p* ≤.05 throughout. Square brackets show the 95% confidence interval around the mean value. For ANOVAs, we report generalized eta squared as a measure of effect size, where a value below 0.06 may be considered small a small effect, a value below 0.14 as medium, and a value equal to or above 0.14 as large (Cohen, 1988).

#### Shooting movements

Because of the simplicity of the shooting movement metrics, we chose to work on the raw cursor time-series data, sampled at the participant’s computer refresh rate. For the majority of participants this refresh rate was 60 Hz, with no significant difference between input devices ($\:W=2818.50,p=.964$). Motion onset was identified as the first frame after target presentation where the cursor was more than 2% of the distance to the target. Reaction times were then the time between target display and motion onset, and movement times were the time between motion onset and the target radius being crossed. We also used the hand angle (or angular error), defined as the difference in angle between the lines joining the start point to the target and the cursor position at the target radius. The cursor position at the target radius was found by linear interpolation between the frame before and after the target radius was crossed.

Trials were removed from the analysis if: the reach was not completed within 300 ms; the hand angle at the target radius was more than 60° either side of the target; or if the hand angle was more than 40° from a moving average of hand angles on recent trials. The former was necessary because long movements were indicative of multiple discrete movements, for example if participants did not reach the target with their initial shooting movement, and the latter two necessary because participants sometimes appeared to predict the next target, initiating movements towards the wrong target. This removed 3% of trials (between-participant SD = 3%).

Because many of the measures studied are known to have skewed distributions (e.g. reaction time), within-subject averages and variabilities were calculated as the median and median absolute deviation (MAD) of trial-level data respectively (apart from the hand angle analysis where, to avoid artificially inflating variability measures in the no feedback group due to large differences in bias across target direction [see Supplementary Fig. 1], medians and MADs respectively were taken within each target direction first and then means calculated over all target directions), whereas group-level averages were calculated as the mean over participants. When calculating MADs, we used a correction factor of 1.4826 to make it a robust estimator of standard deviation. Specifically, we analysed average reaction time, average movement time, average hand angle (bias), and variability in hand angle as outcome measures. ANOVAs to assess main effects of feedback type (with and without visual feedback) and input device (mouse and trackpad), and their interaction, on these outcome variables were conducted using the *afex* package. Where main effects (to identify average values per grouping variable) or interactions (to understand whether differences were restricted to certain comparisons) were followed up, estimated marginal means were calculated and compared using the *emmeans* package, with Bonferroni-Holm corrections applied where more than one comparison was performed.

Because of the significant difference in gender proportions per input device in this experiment, we conducted additional analyses where we included gender (restricted to male or female for statistical power) in all ANOVAs. We found that the significance of all effects involving input device did not change with the inclusion of gender. We found a single analysis showing significant effects including gender, where the bias in hand angle showed an interaction of input device and gender and an interaction of input device, feedback group, and gender, but post-hoc comparisons indicated neither within-device comparison was significant. We also ran an exploratory analysis demonstrating the risk that ignoring input device can have. We performed two linear regressions with reaction time as the dependent variable, one where only gender was entered as a predictor (again restricted to males and females), and another where both gender and input device were entered as predictors.

#### Point-to-point movements

We processed the data for this experiment per the original work (Warburton et al., 2023a). Cursor position timeseries data was resampled from the participant’s screen refresh rate (the majority had a refresh rate of 60 Hz, with no significant difference between input device, $\:W=807.5,p=.892$) to 100 Hz using linear interpolation, and filtered using a second order, low-pass Butterworth filter with a 15 Hz cut-off in the forward and reverse directions to give zero lag. Individual movements within each round were segmented using the time between the previous and current target being clicked. Radial speed, acceleration, and jerk data were obtained by numerical differentiation of the cursor radius (*pracma* package) after centring the cursor against the start-point of each movement.

The total time between successfully clicking the previous and current target gave the acquire time, which was decomposed into a number of other temporal metrics. Reaction time was defined as the time between the previous target being successfully clicked and motion onset, where radial speed first rose above 0.5 au/s, and the movement time was defined as the time between motion onset and the target being clicked. The movement time could be further broken down into the primary movement time, the correction time, and the click dwell time. The primary movement time measured the time between motion onset and the end of the primary movement, where discrete feedback corrections supposedly start (Elliott et al., 2010). We calculated the end of the primary movement using a similar procedure to other papers (Abrams & Pratt, 1993; Meyer et al., 1988) by looking for any of the following criteria after peak radial speed: the radial speed fell below 0.5 au/s, indicating movement termination or upcoming direction reversal; the radial acceleration crossed from negative to positive, indicating participants increased their speed; or the radial jerk cross from positive to negative, indicating participants were ‘braking’. The correction time measured the time between the end of the primary movement and the target being entered, representing the time required to execute corrections whereafter a trial could be ended. The click dwell time measured the time between the target being entered and a successful click being registered. Note that, because medians were used to calculate within-subject averages, the summation of the group averages for these measures does not equal the group average for acquire time.

For the spatial metrics, we calculated average radial extent and speed per timestep to understand the progression of these variables over the trial. From the radial speed data, we extracted the peak radial speed reached. We extracted further spatial measures at 100 ms into the movement to provide comparisons at a consistent time-point. These included average and variability in both the proportion of the target distance covered (extent in the target direction divided by target distance) and the hand angle. We also calculated two measures capturing whole-movement properties. The linearity index should complement the angular variability analysis as it measures how far a given movement deviated from the ideal path, with values closer to zero indicating lower curvature (the absolute maximum perpendicular deviation from a straight line joining the start point and the target centre divided by that line’s length; Sergio and Scott (1998). Further, the log dimensionless jerk captures how smooth movements are, with values closer to zero indicating smoother movements (the natural logarithm of the summed squared jerk normalised by the ratio of the movement duration cubed by the peak speed squared; Balasubramanian et al. (2012). A maximally efficient movement minimising jerk produces a speed profile with a single symmetrical peak (Flash & Hogan, 1985). Less efficient movements, for example those with additional accelerations in the speed profile or entirely distinct sub-movements, will be reflected in a more negative log dimensionless jerk.

For this experiment we only removed movements if the hand angle at 100 ms into a movement was more than 60° from the target angle, removing 3% of movements (between-participant SD = 3%). Evidently, participants still occasionally executed ill-directed movements even with the following target visible. In addition, 1 round of data was not uploaded for 1 participant. As in the shooting movement experiment, we calculated within-subject averages and variabilities as the median and median absolute deviation (with correction factor) respectively, whereas group-level averages were calculated as the mean. An ANOVA (Greenhouse-Geisser sphericity corrections applied) showed participants improved their acquire time over the experiment (the participant with a missing round of data was excluded from this single analysis), with visual inspection suggesting this had mostly plateaued by the last 10 rounds, so further analysis was done on data collapsed over the last 10 rounds only. For comparisons of discrete outcomes (e.g. reaction times) we used t-tests (equal variance assumed). For comparisons of continuous outcomes (e.g. radial speed profiles) we used cluster-based permutation tests to identify clusters of significant differences (Maris & Oostenveld, 2007) using the *jlmerclusterperm* library (t-statistic threshold of 2.5, 1000 simulations).

## Electronic supplementary material

Below is the link to the electronic supplementary material.

Supplementary Material 1

## Data Availability

The data and code used will be available upon publication on the Open Science Framework, at https://osf.io/4m8hb/.
