# Supplementary material for: Input device matters for measures of behaviour in online experiments
Source: Psychol Res. 2024 Nov 28;89(1):29. doi: 10.1007/s00426-024-02065-1 (PMC11604694; doi:10.1007/s00426-024-02065-1)
Supplement: Supplementary file 1 — Supplementary Material 1 [file 426_2024_2065_MOESM1_ESM.pdf]

Supplementary materials for: Input device matters for measures of behaviour in online experiments

Matthew Warburton[1], Carlo Campagnoli[1], Mark Mon-Williams[1,2,3,4], Faisal Mushtaq[1,4,5], and J. Ryan Morehead[1,5]
[1]School of Psychology, University of Leeds, UK
[2]Bradford Institute for Health Research, Bradford Hospitals National Health Service Trust, UK
[3]National Centre for Optics, Vision and Eye Care, University of South-Eastern Norway, Norway
[4]NIHR Leeds Biomedical Research Centre, UK
[5]Centre for Immersive Technologies, University of Leeds, UK

Angle bias and variability across target directions.

In addition to differences in the average angular bias and variability between input devices, we found large heterogeneity in both measures across target angle (Supplementary Figure 1). The effect of target direction on angular biases and their amelioration with feedback is well documented (Ghilardi et al., 1995), and recent work demonstrates these patterns differ slightly across arm and trackpad movements (Wang et al., 2024). These patterns also differ between mouse and trackpad movements, in particular when no visual feedback of movements is provided. The peak-to-peak magnitude (difference in maximum vs minimum bias) for users with no feedback was 25.0° for trackpad users and 11.2° for mouse users, which reduced to 3.9° and 3.6° respectively when visual feedback was provided. We found main effects of input device ($F_{1,149} = 35.60, \eta_g^2 = .06, p < .001$) and target angle ($F_{7.58,1128.89} = 44.03, \eta_g^2 = .18, p < .001$), but no main effect of feedback ($F_{1,149} = 0.02, \eta_g^2 < .01, p = .896$). All two-way interactions ($F > 14.04, \eta_g^2 > .03, p's < .001$) and the three-way interaction ($F_{7.58,1128.89} = 11.36, \eta_g^2 = .05, p < .001$) were also significant. Note that Greenhouse-Geisser sphericity corrections were applied for terms including target direction. Consistent with previous work (Wang et al., 2024) we found that the bias functions correlated well within input device across groups (Mouse: Pearson's $r_{22} = .57, p = .003$ r_22 = .57,p = .003; Trackpad: $r_{22} = .83, p < .001$).

We also found a distinct pattern of variability across target directions both with and without visual feedback, where variability appeared to reach local minima for cardinal directions. We found main effects of input device ($F_{1,149} = 7.87, \eta_g^2 = .02, p = .006$), feedback ($F_{1,149} = 25.31, \eta_g^2 = .06, p < .001$), and target angle ($F_{17.89,2665.47} = 9.72, \eta_g^2 = .04, p < .001$). There was no interaction between input device and pointer ($F_{1,149} = 0.00, \eta_g^2 < .01, p = .973$), but the other two-way interactions ($F > 2.65, \eta_g^2 > .01, p's < .001$) and the three-way interaction ($F_{17.89,2665.47} = 1.82, \eta_g^2 = .01, p = .018$) were significant. Note that Greenhouse-Geisser sphericity corrections were applied for terms including target direction.

References

Ghilardi, M. F., Gordon, J., & Ghez, C. (1995). Learning a visuomotor transformation in a local area of work space produces directional biases in other areas. *Journal of Neurophysiology*, *73*(6), 2535–2539. https://doi.org/10.1152/jn.1995.73.6.2535

Wang, T., Morehead, R. J., Tsay, J. S., & Ivry, R. B. (2024, March 18). *The Origin of Movement Biases During Reaching*. https://doi.org/10.1101/2024.03.15.585272

Correspondence should be addressed to Matthew Warburton, Email: pscmwa@leeds.ac.uk

Supplementary Figure 1

Differences in bias and variability in hand angle across direction.

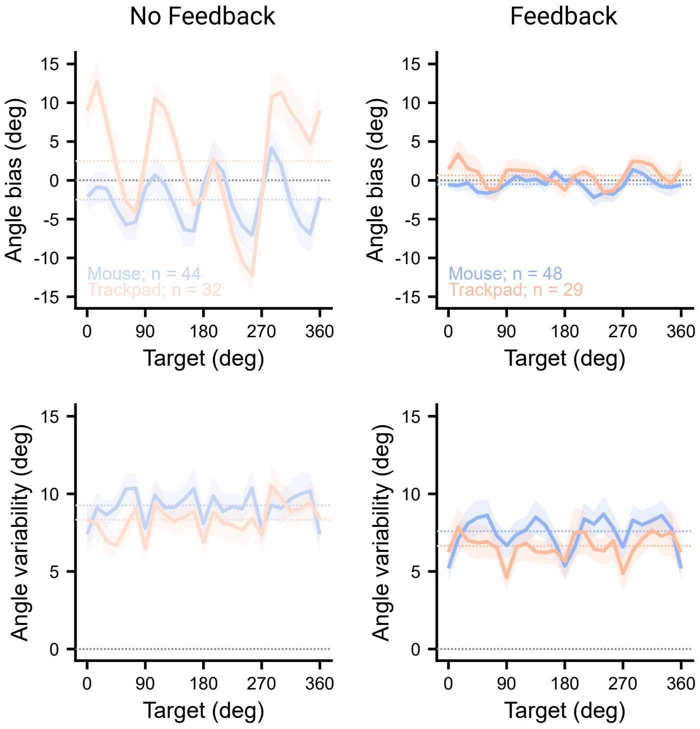

Note. A target angle of 0° is directly to the right of the start point, with positive angles going counter-clockwise.
